# Supplementary material for: Transcriptomic and Physiological Responses to Oxidative Stress in a Chlamydomonas reinhardtii Glutathione Peroxidase Mutant
Source: Genes (Basel). 2020 Apr 24;11(4):463. doi: 10.3390/genes11040463 (PMC7230881; doi:10.3390/genes11040463)
Supplement: Supplementary file 1 [file genes-11-00463-s001.zip › Supplementary Files/supplementary figure+legends.pptx]

## Slide 1
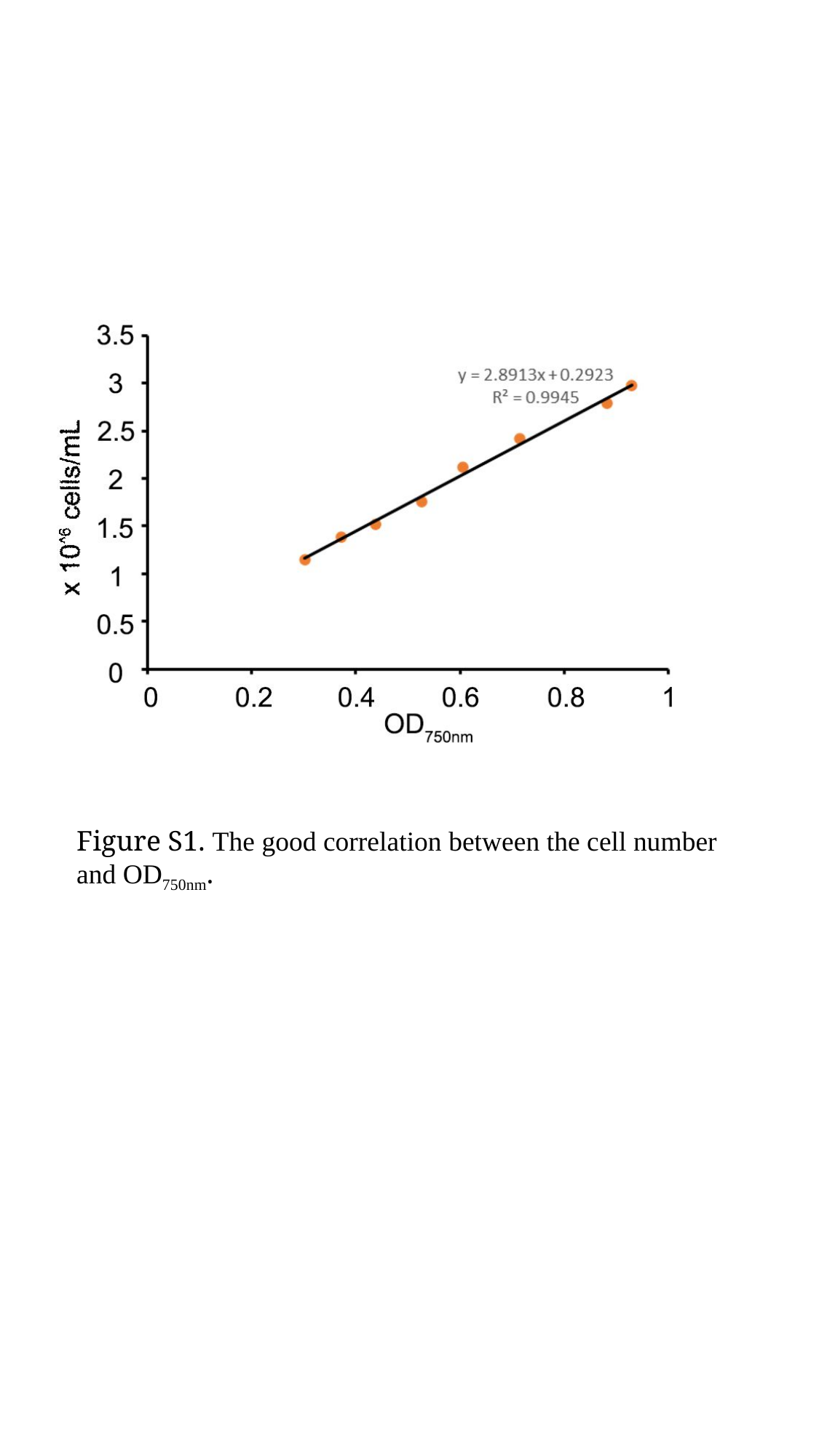

Figure S1. The good correlation between the cell number and OD750nm.

## Slide 2
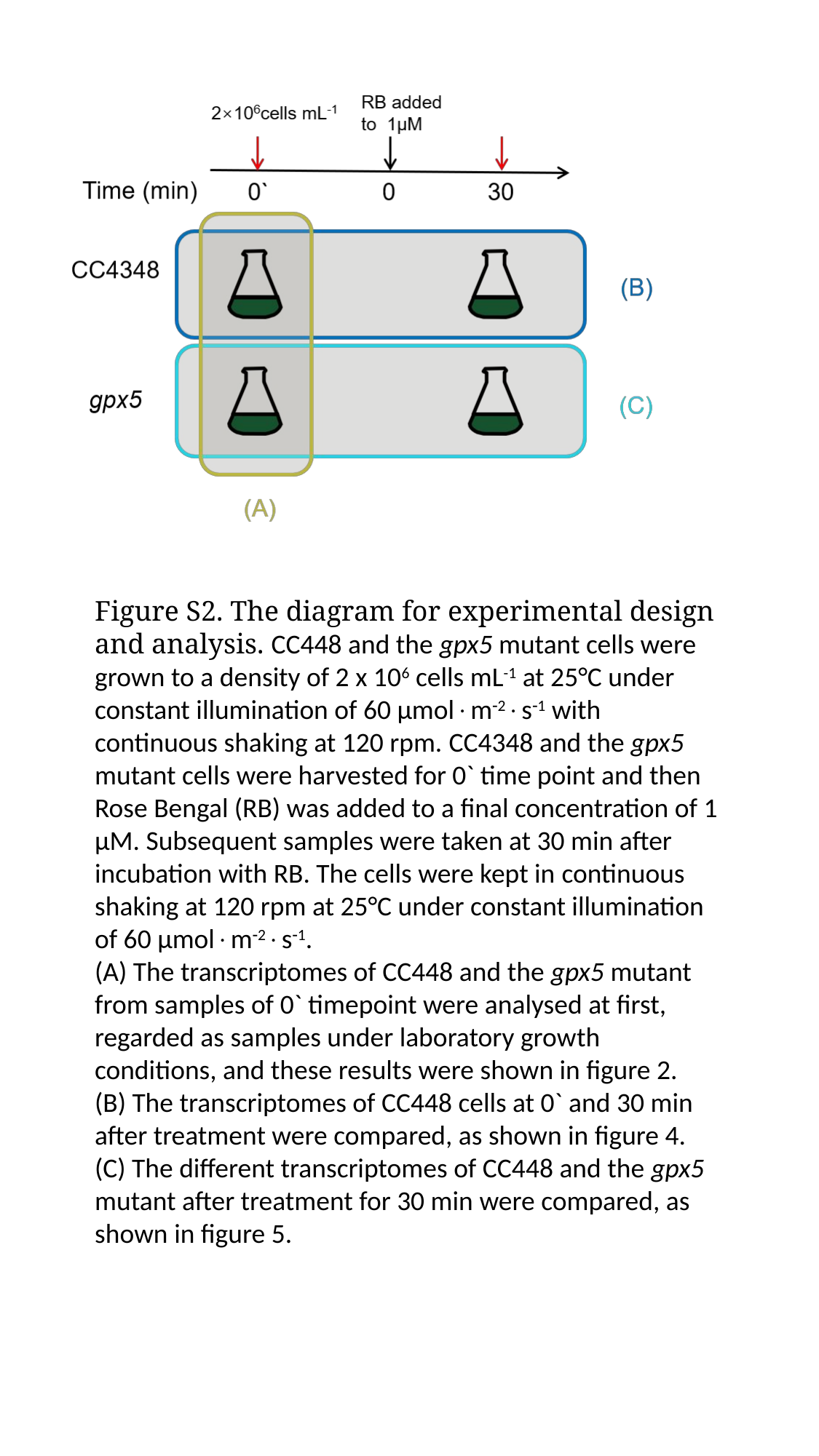

Figure S2. The diagram for experimental design and analysis. CC448 and the gpx5 mutant cells were grown to a density of 2 x 10^6^ cells mL-1 at 25°C under constant illumination of 60 µmolm−2s−1 with continuous shaking at 120 rpm. CC4348 and the gpx5 mutant cells were harvested for 0` time point and then Rose Bengal (RB) was added to a final concentration of 1 µM. Subsequent samples were taken at 30 min after incubation with RB. The cells were kept in continuous shaking at 120 rpm at 25°C under constant illumination of 60 µmolm−2s−1.
(A) The transcriptomes of CC448 and the gpx5 mutant from samples of 0` timepoint were analysed at first, regarded as samples under laboratory growth conditions, and these results were shown in figure 2.
(B) The transcriptomes of CC448 cells at 0` and 30 min after treatment were compared, as shown in figure 4.
(C) The different transcriptomes of CC448 and the gpx5 mutant after treatment for 30 min were compared, as shown in figure 5.

## Slide 3
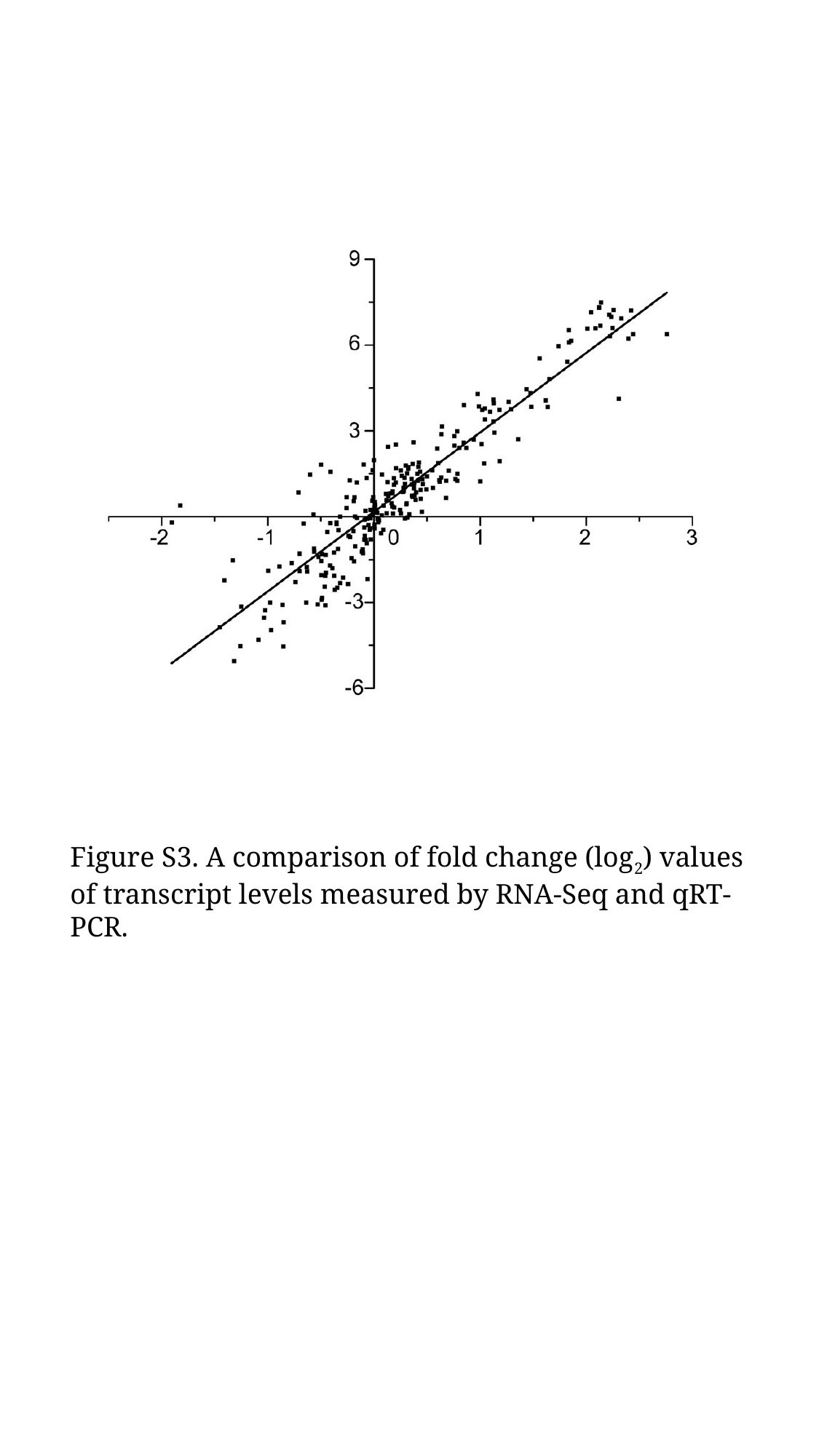

Figure S3. A comparison of fold change (log2) values of transcript levels measured by RNA-Seq and qRT-PCR.

## Slide 4
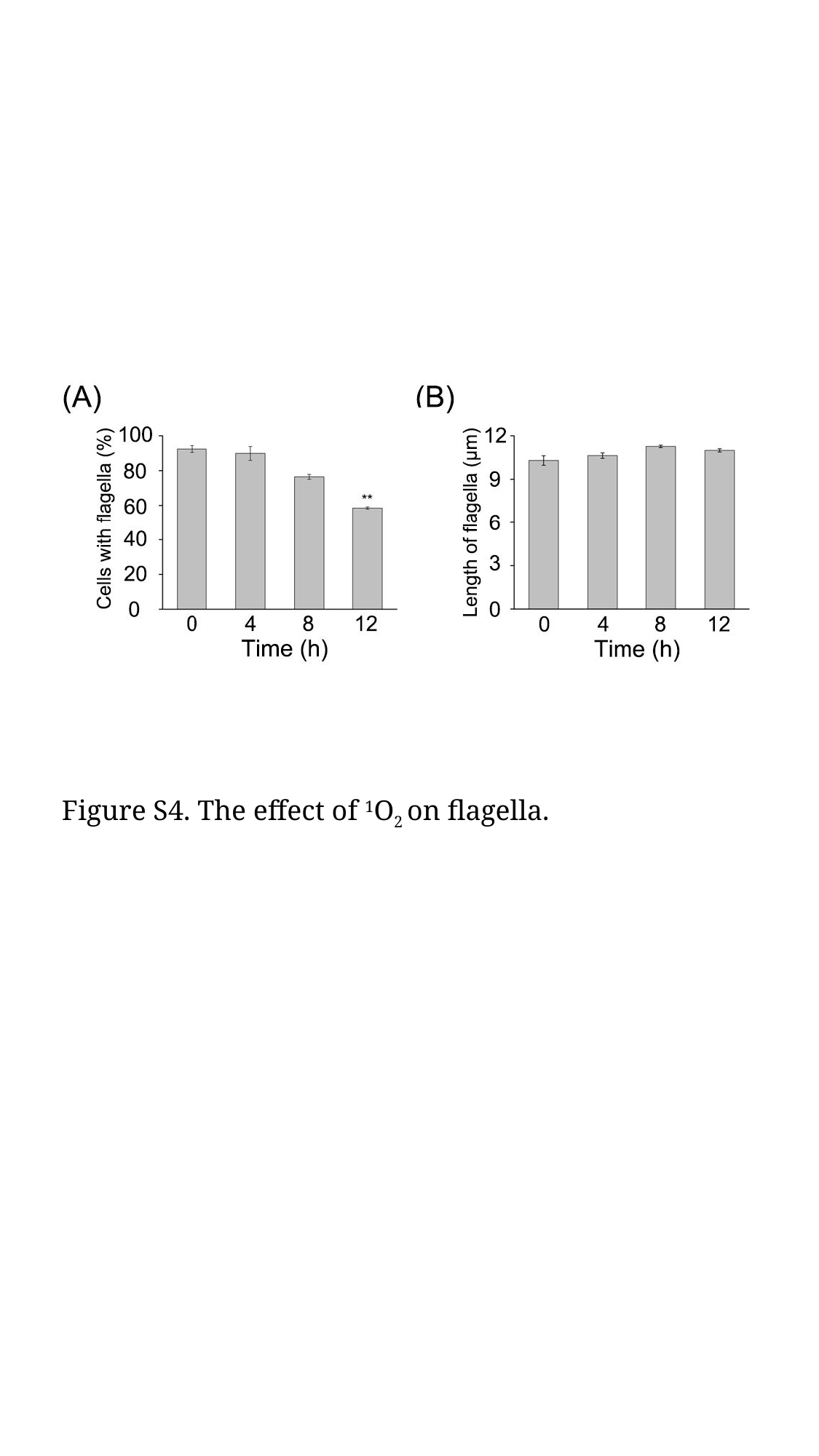

Figure S4. The effect of 1O2 on flagella.

## Slide 5
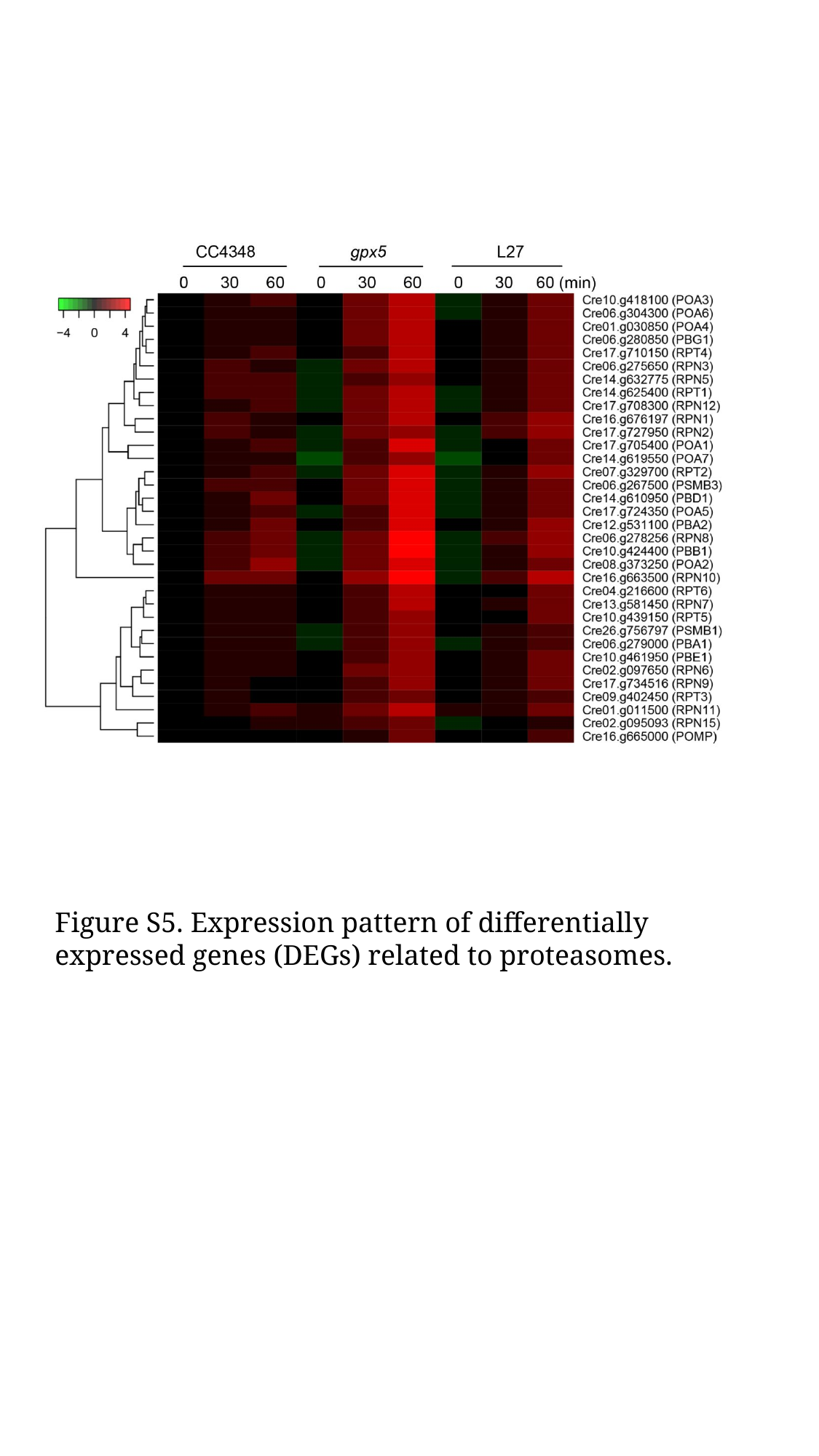

Figure S5. Expression pattern of differentially expressed genes (DEGs) related to proteasomes.

## Slide 6
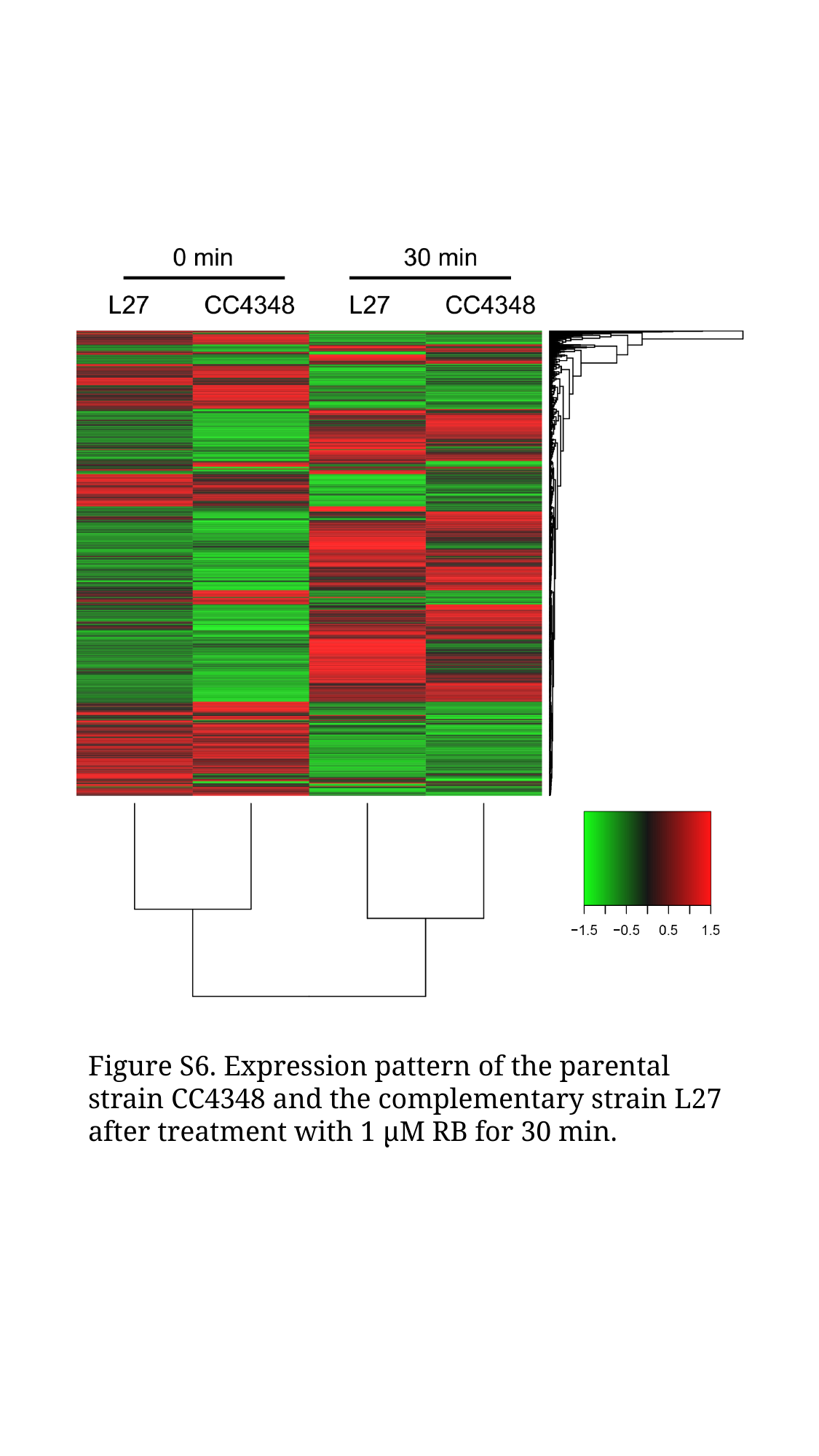

Figure S6. Expression pattern of the parental strain CC4348 and the complementary strain L27 after treatment with 1 μM RB for 30 min.

## Slide 7
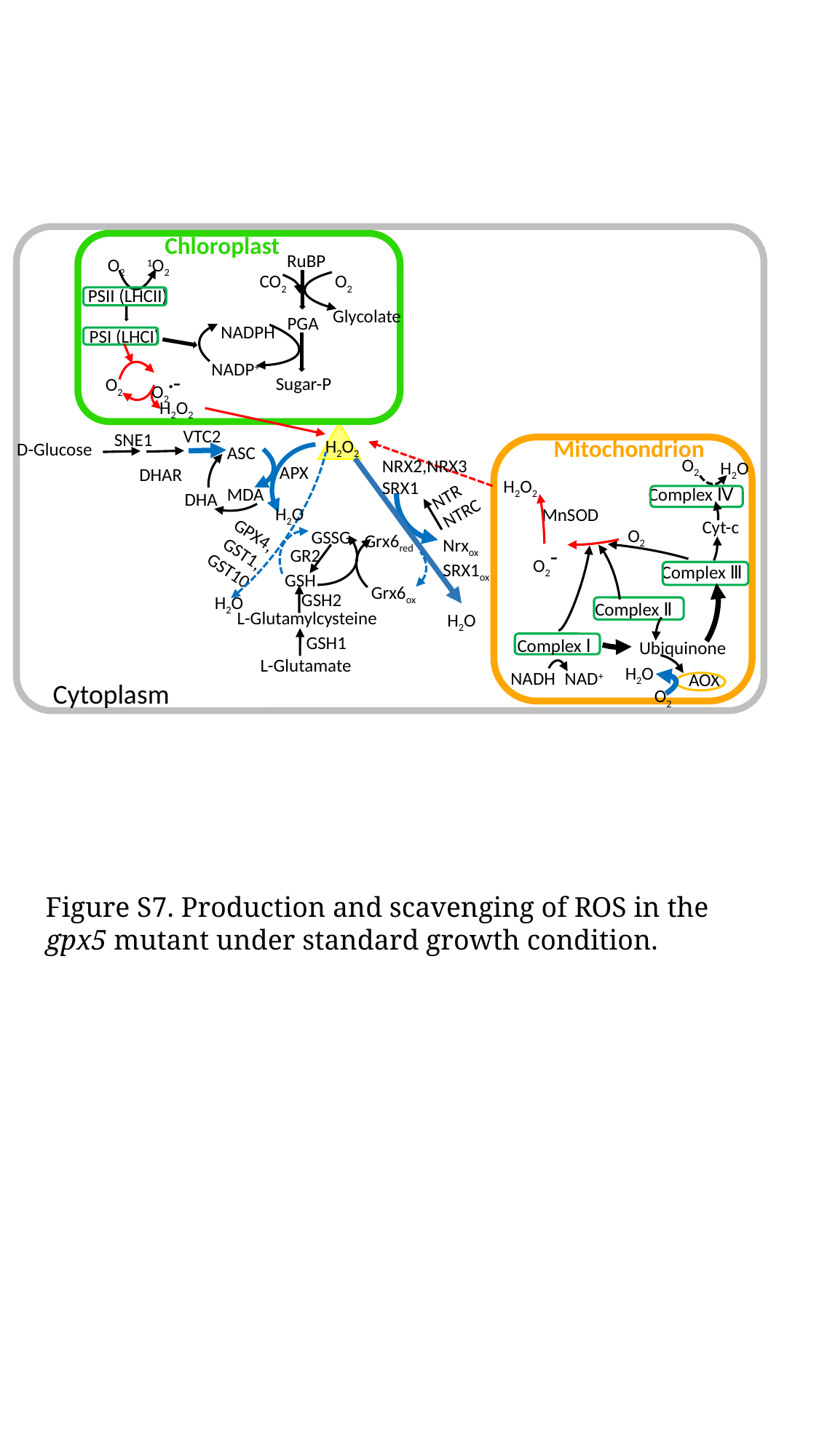

Chloroplast
RuBP
O2
1O2
CO2
O2
PSII (LHCII)
Glycolate
PGA
NADPH
PSI (LHCI)
O2●-
NADP+
Sugar-P
O2
H2O2
VTC2
SNE1
Mitochondrion
H2O2
D-Glucose
ASC
O2
NRX2,NRX3
SRX1
H2O
APX
DHAR
H2O2
NTR
NTRC
MDA
Complex Ⅳ
DHA
H2O
MnSOD
Cyt-c
GPX4,
GST1,
GST10
O2
GSSG
Grx6red
O2-
Nrxox
SRX1ox
GR2
Complex Ⅲ
GSH
Grx6ox
GSH2
H2O
Complex Ⅱ
L-Glutamylcysteine
H2O
GSH1
Complex Ⅰ
Ubiquinone
L-Glutamate
H2O
NADH
NAD+
AOX
Cytoplasm
O2
Figure S7. Production and scavenging of ROS in the gpx5 mutant under standard growth condition.

## Slide 8
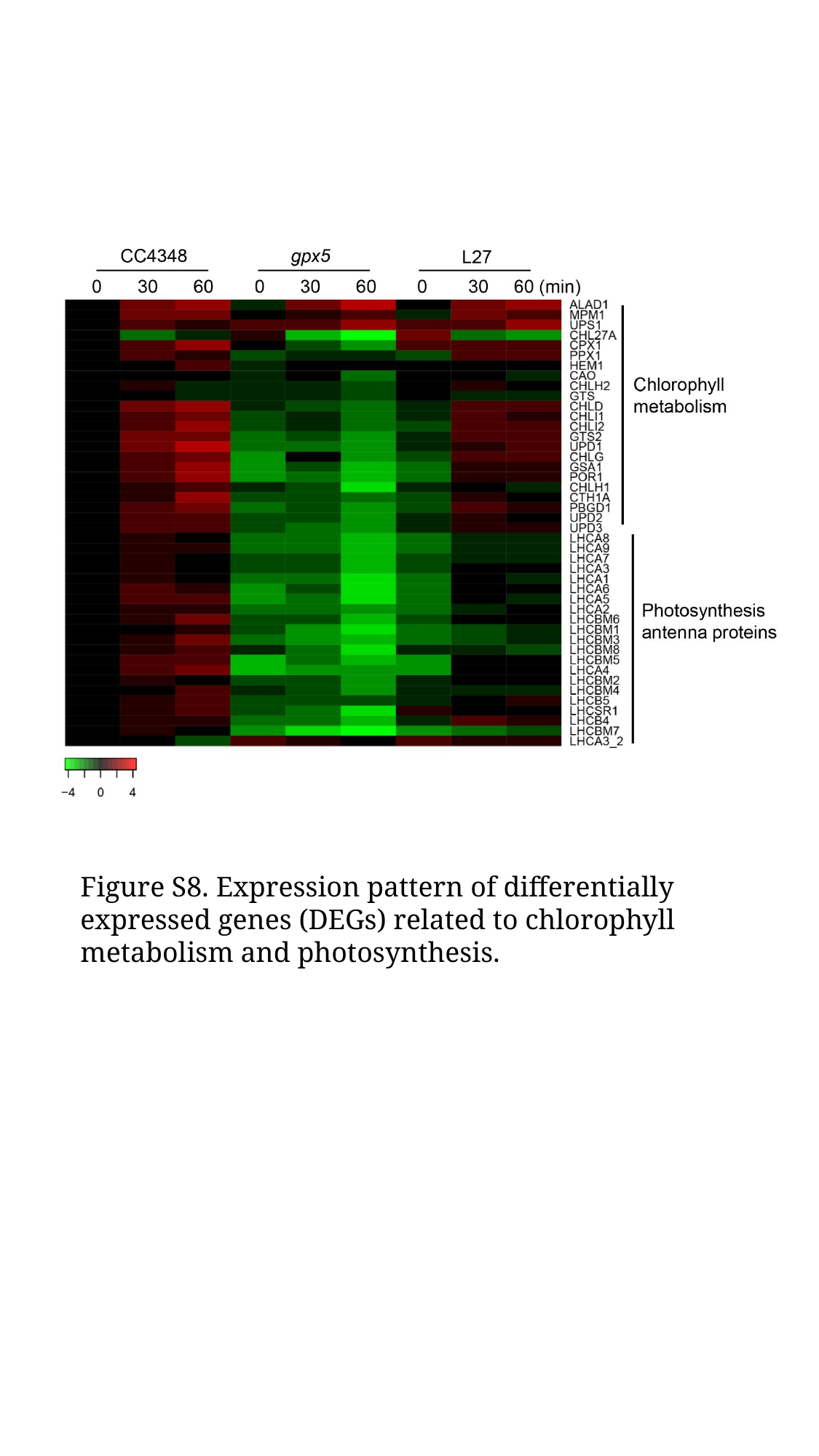

Figure S8. Expression pattern of differentially expressed genes (DEGs) related to chlorophyll metabolism and photosynthesis.

## Slide 9
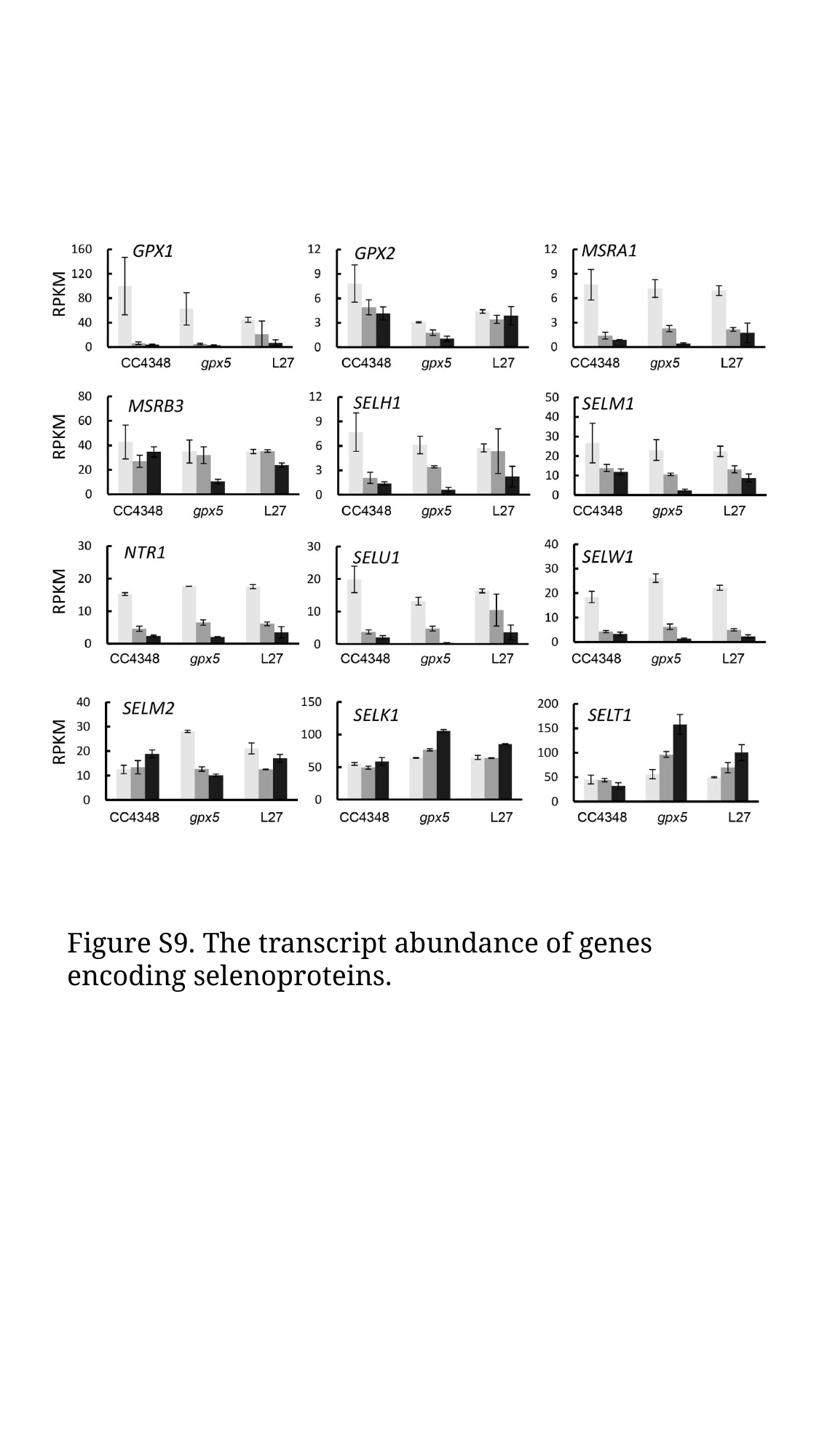

Figure S9. The transcript abundance of genes encoding selenoproteins.
