# Supplementary figures and images for: Transcriptomic and Physiological Responses to Oxidative Stress in a Chlamydomonas reinhardtii Glutathione Peroxidase Mutant
Source: Genes (Basel). 2020 Apr 24;11(4):463. doi: 10.3390/genes11040463 (PMC7230881; doi:10.3390/genes11040463)

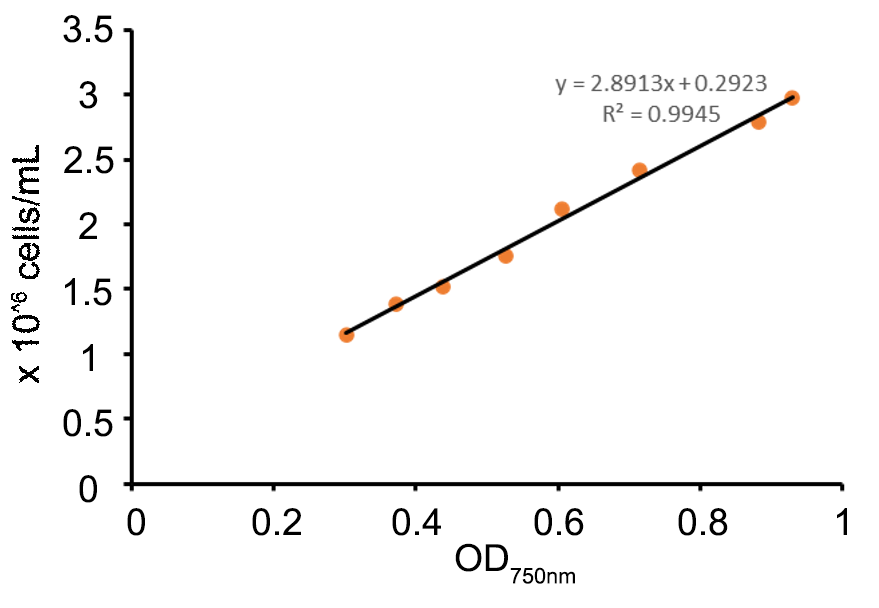

Supplement: Supplementary file 1 [file genes-11-00463-s001.zip › Supplementary Files/Figure S1.tif]

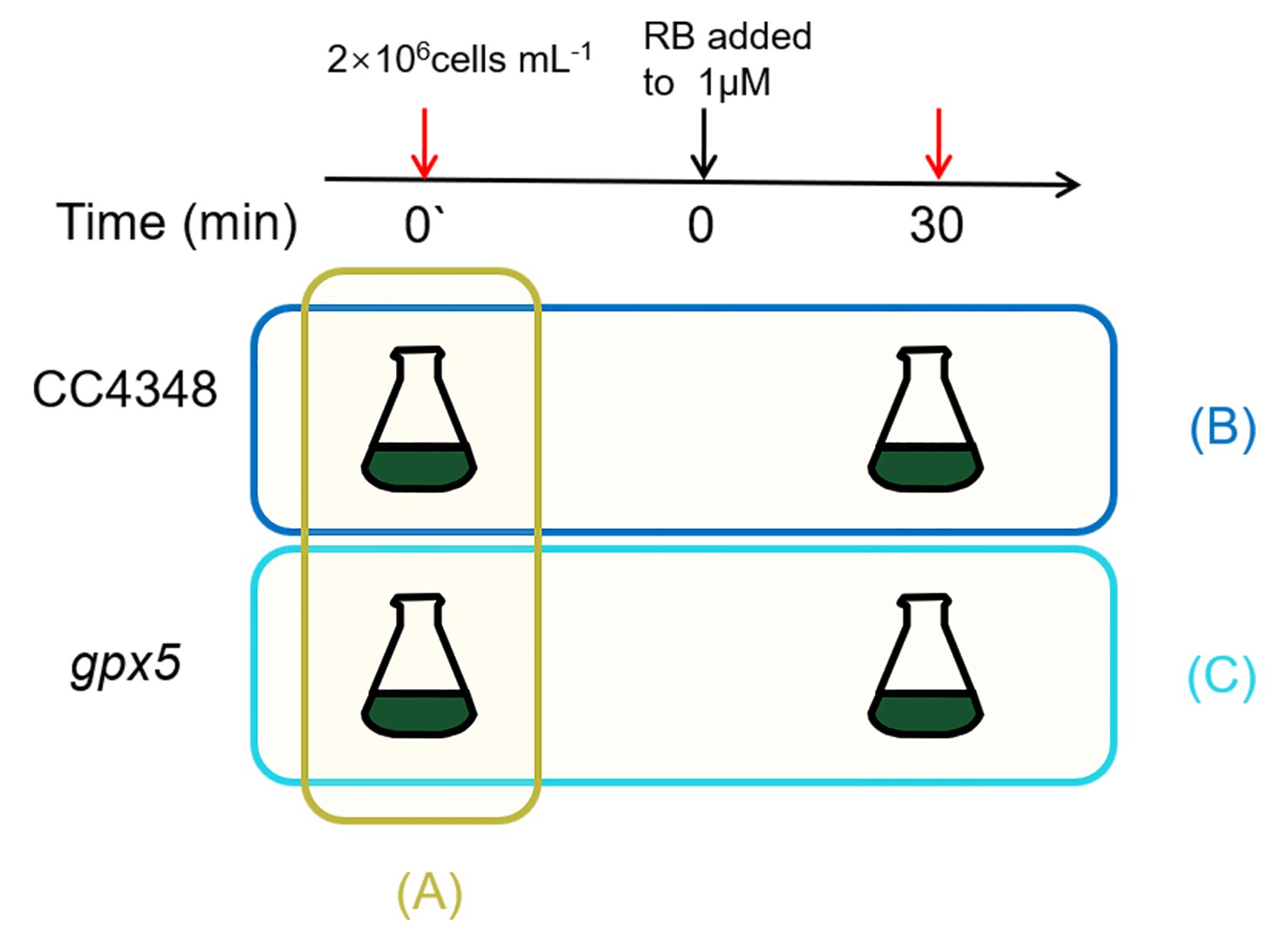

Supplement: Supplementary file 1 [file genes-11-00463-s001.zip › Supplementary Files/Figure S2.tif]

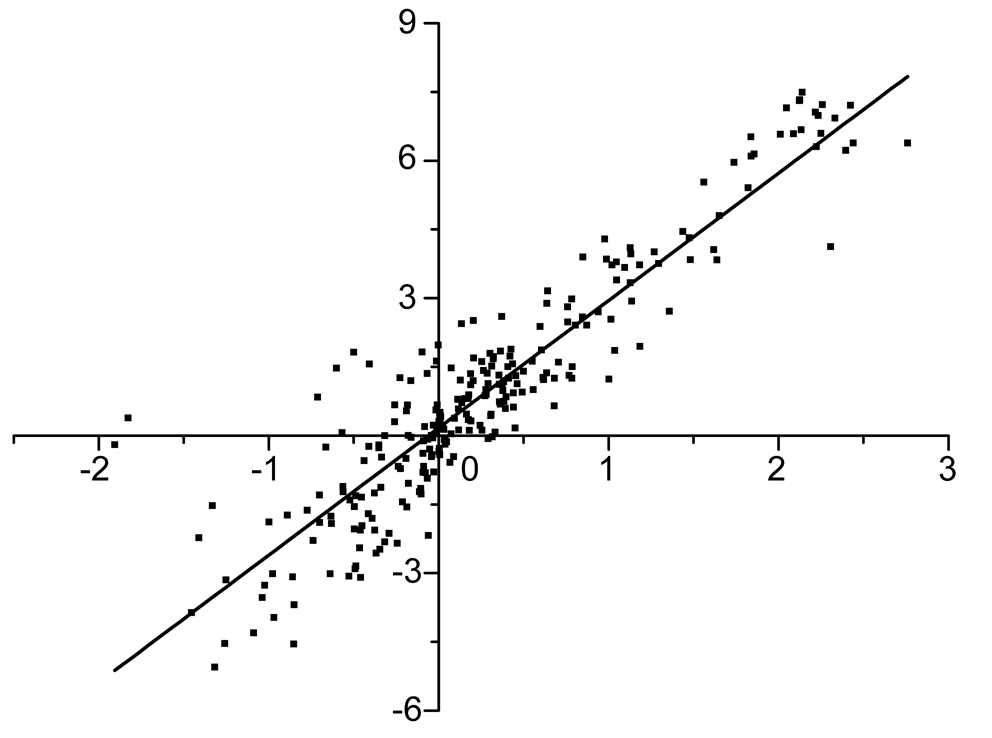

Supplement: Supplementary file 1 [file genes-11-00463-s001.zip › Supplementary Files/Figure S3.tif]

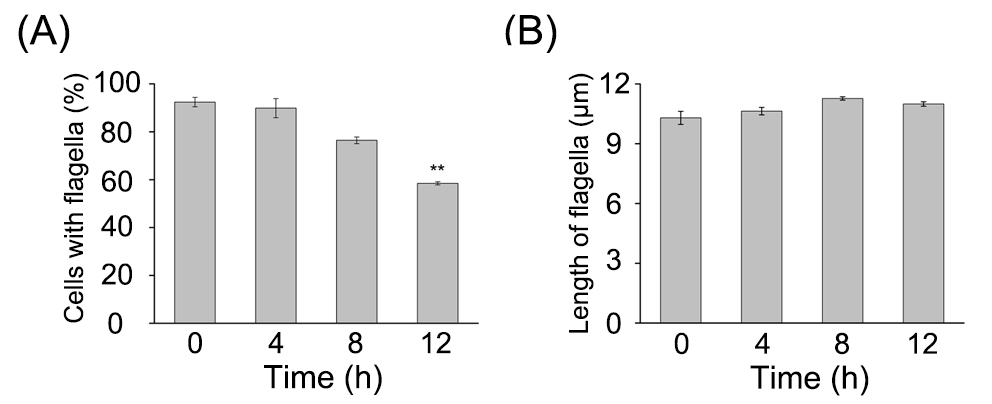

Supplement: Supplementary file 1 [file genes-11-00463-s001.zip › Supplementary Files/Figure S4.tif]

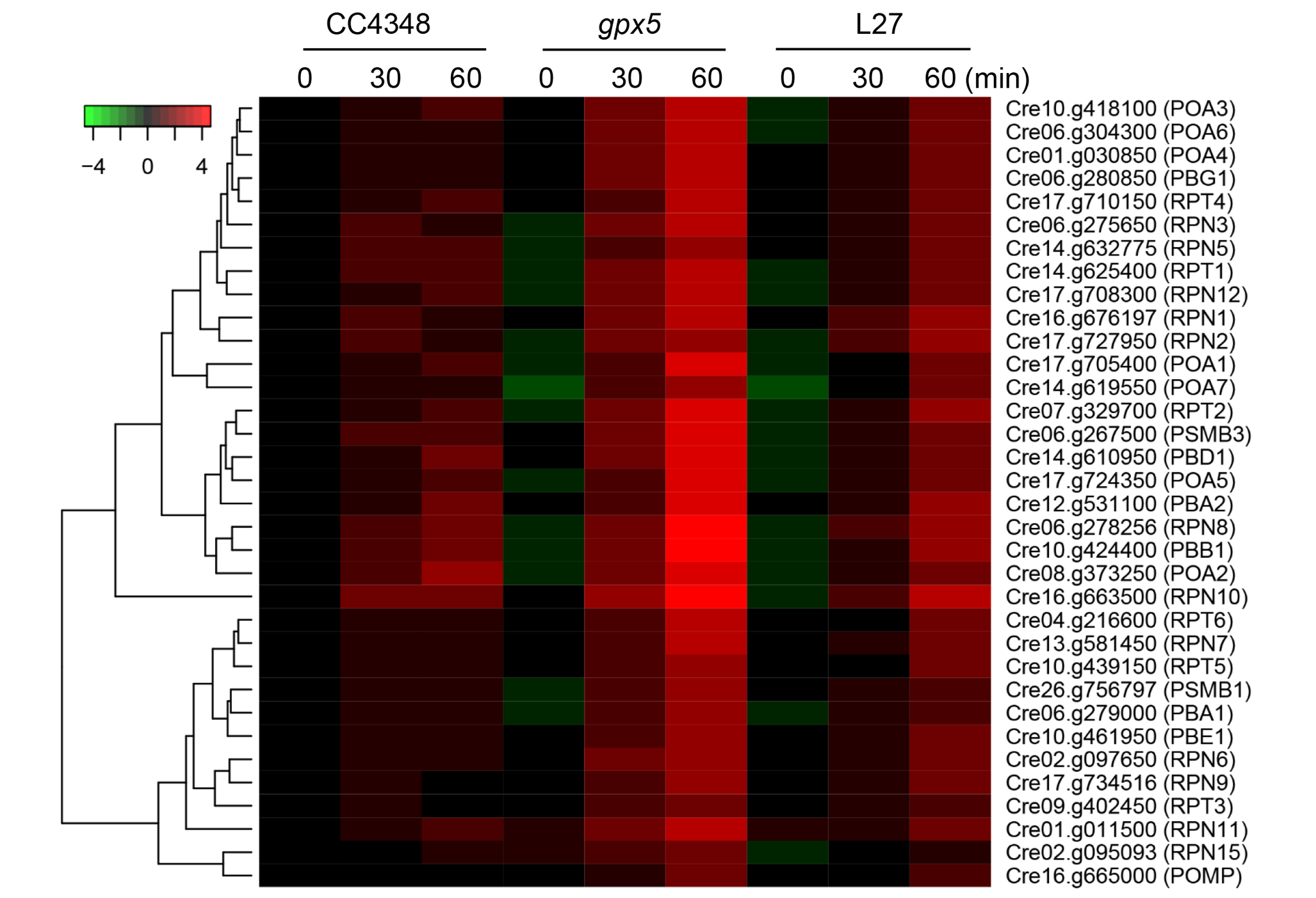

Supplement: Supplementary file 1 [file genes-11-00463-s001.zip › Supplementary Files/Figure S5.tif]

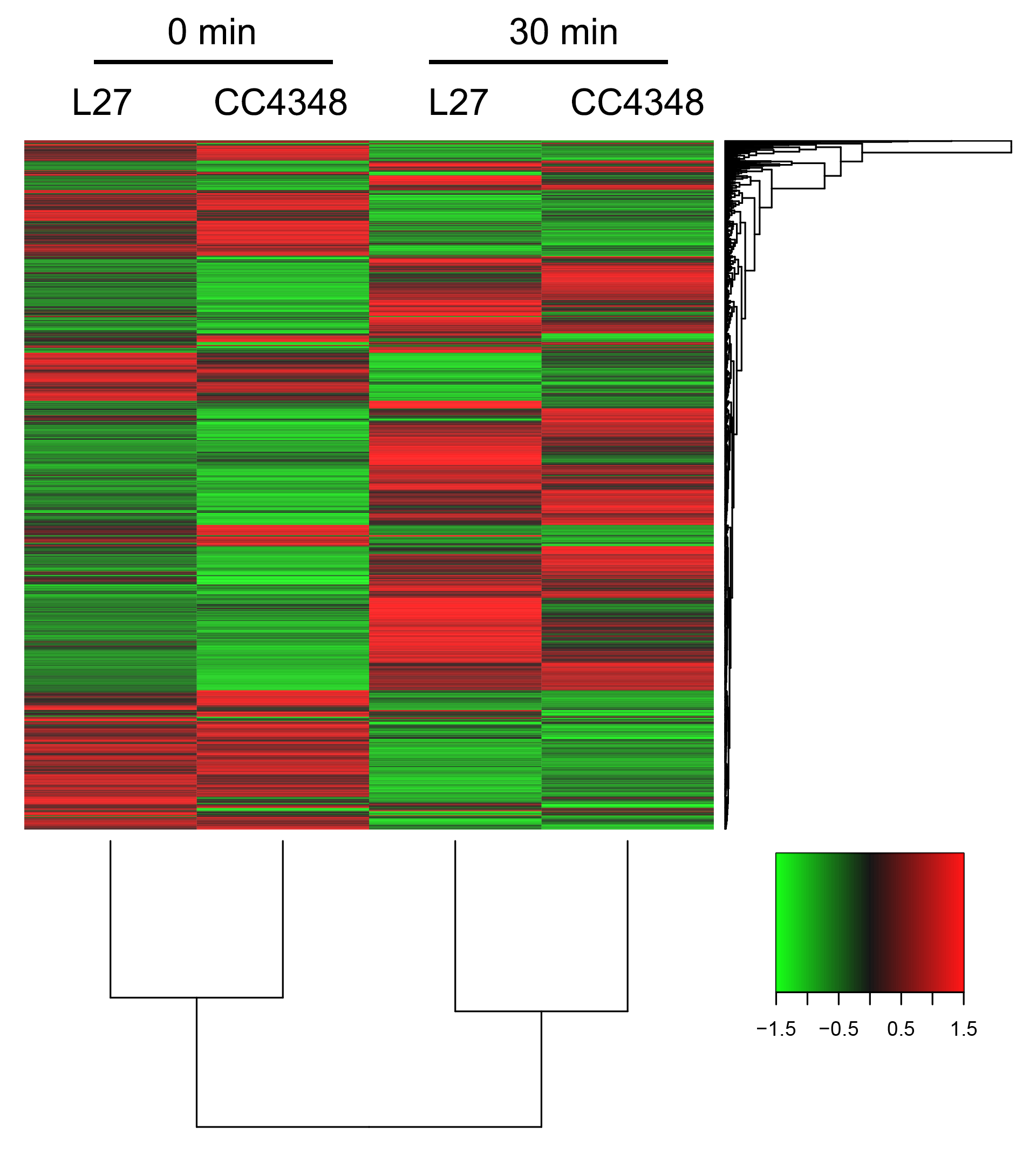

Supplement: Supplementary file 1 [file genes-11-00463-s001.zip › Supplementary Files/Figure S6.tif]

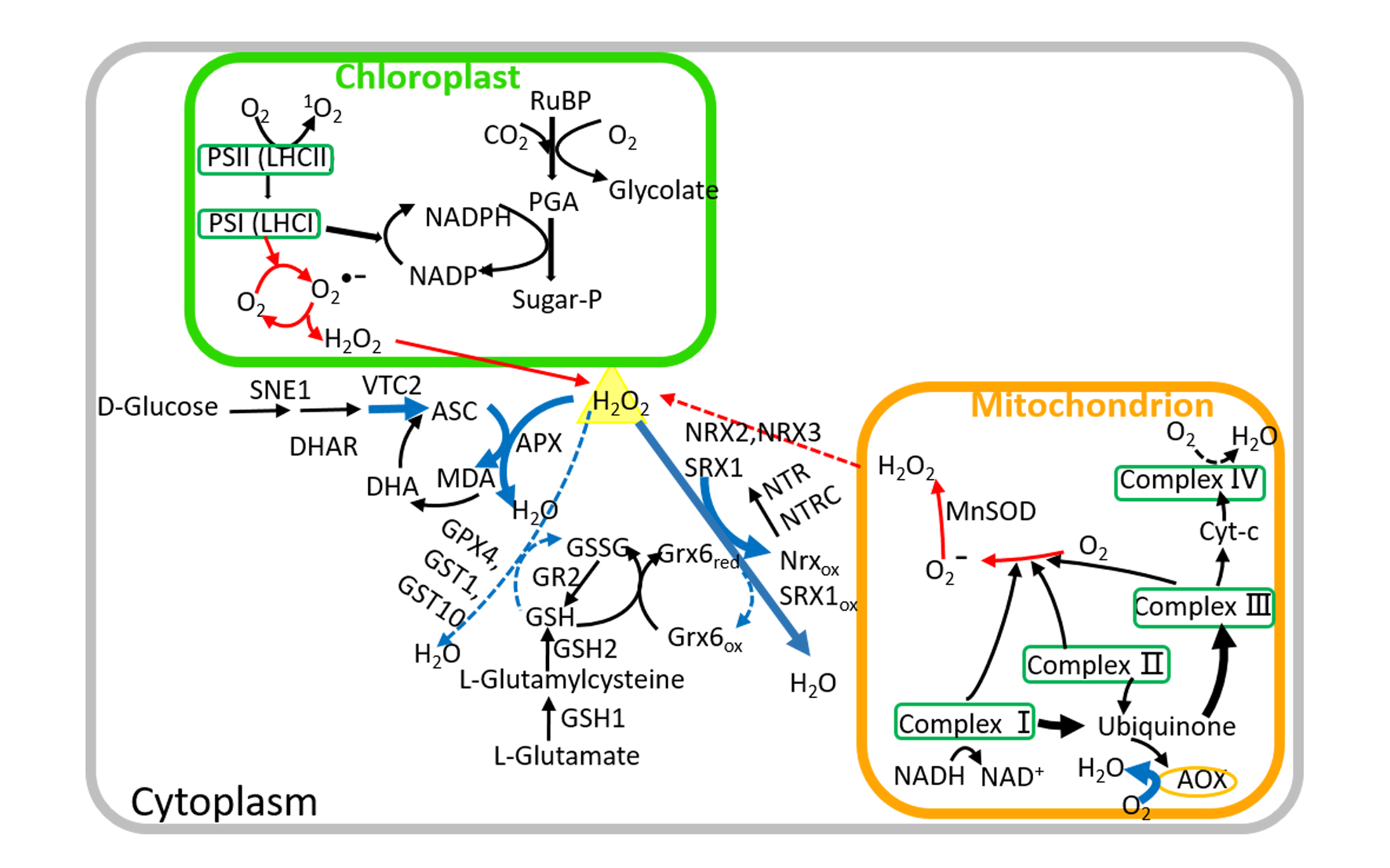

Supplement: Supplementary file 1 [file genes-11-00463-s001.zip › Supplementary Files/Figure S7.tif]

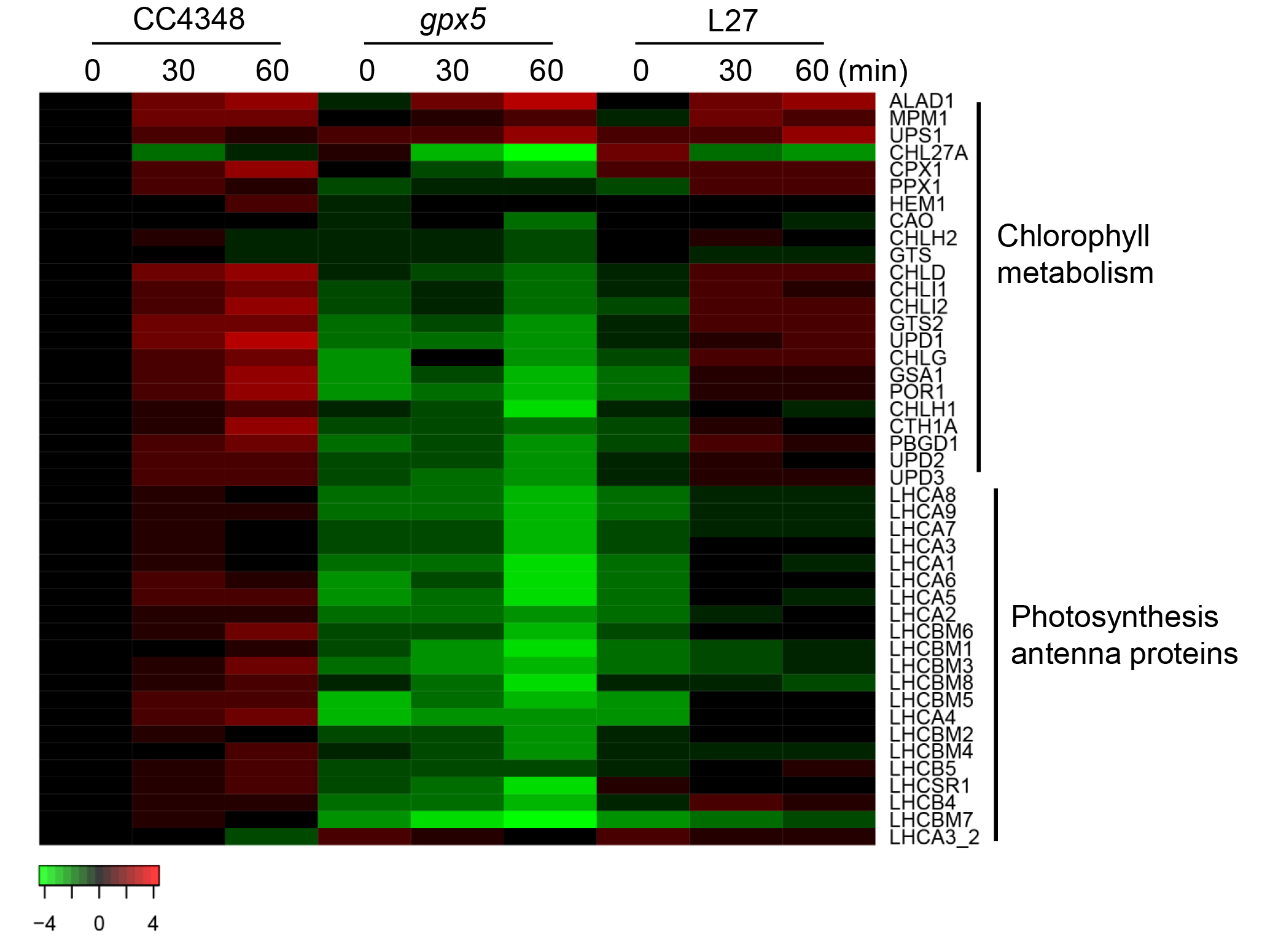

Supplement: Supplementary file 1 [file genes-11-00463-s001.zip › Supplementary Files/Figure S8.tif]

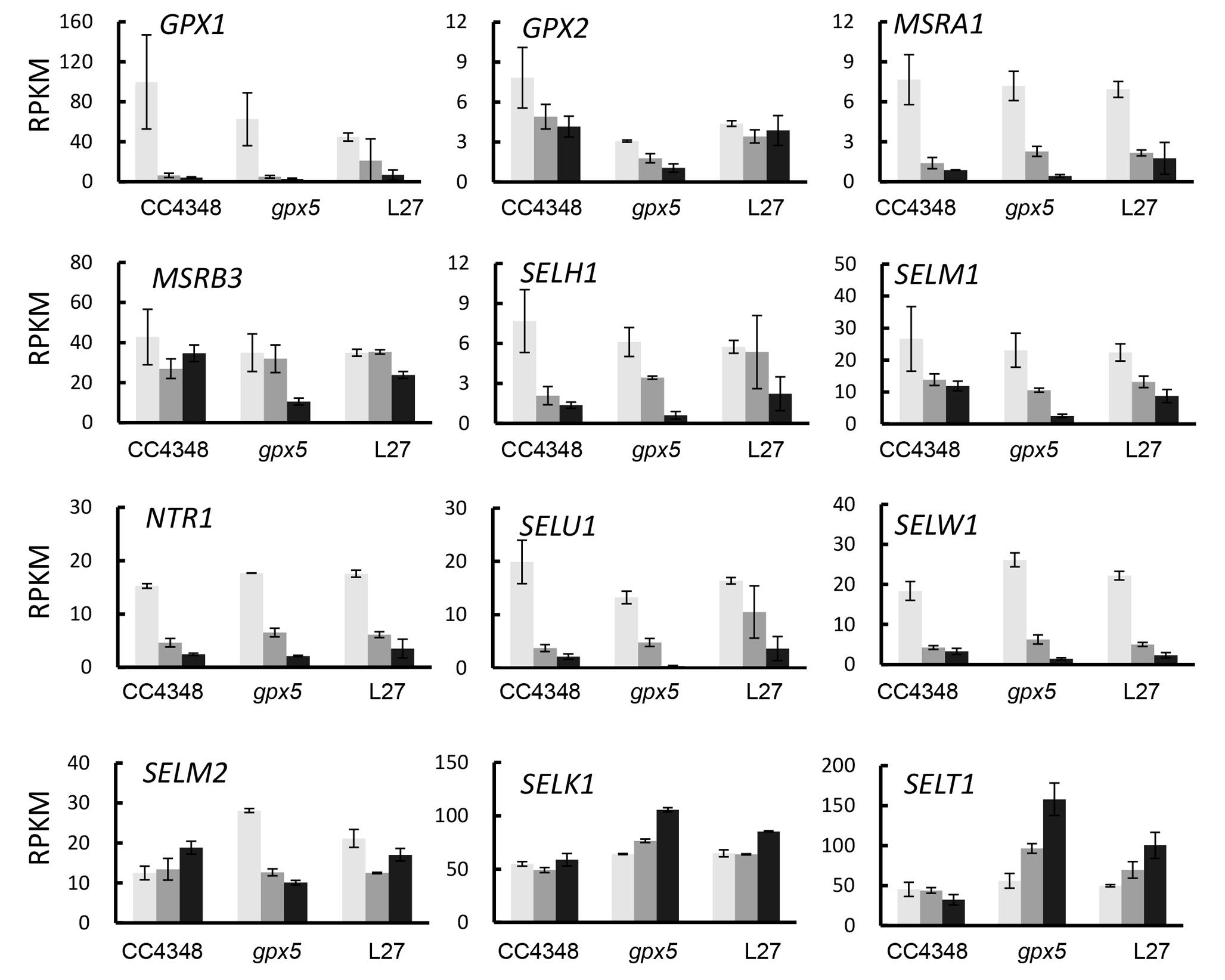

Supplement: Supplementary file 1 [file genes-11-00463-s001.zip › Supplementary Files/Figure S9.tif]
